# Effects of prenatal Poly I:C exposure on global histone deacetylase (HDAC) and DNA methyltransferase (DNMT) activity in the mouse brain

**DOI:** 10.1007/s11033-016-4006-y

**Published:** 2016-05-23

**Authors:** Yara Pujol Lopez, Gunter Kenis, Waldtraud Stettinger, Karin Neumeier, Sylvia de Jonge, Harry W. M. Steinbusch, Peter Zill, Daniel L. A. van den Hove, Aye M. Myint

**Affiliations:** School for Mental Health and Neuroscience (MHeNS), Department of Psychiatry and Neuropsychology, Maastricht University Medical Centre+, Universiteitssingel 50, Room 1.148, 6229 ER Maastricht, The Netherlands; Laboratory of Translational Neuroscience, Department of Psychiatry, Psychosomatics and Psychotherapy, University of Würzburg, Würzburg, Germany; Psychiatric Hospital, Ludwig-Maximilian University, Munich, Germany

**Keywords:** Infection, Pregnancy, HDAC, Epigenetics, Mouse, Poly I:C

## Abstract

The aim of our study was to investigate the brain-specific epigenetic effects on global enzymatic histone deacetylase (HDAC) and DNA methyltransferase (DNMT) activity after prenatal exposure to maternal immune challenge by polyinosinic:polycytidylic acid (Poly I:C) at gestational day (GD) 17 in C57BL/6JRccHsd mouse offspring. Pregnant mice were randomly divided into 2 groups, receiving either 5 mg/kg Poly I:C or phosphate buffered saline (PBS) intravenously at GD 17. Subsequently, the effects on whole brain enzymatic HDAC and DNMT activity and the protein levels of various HDAC isoforms were assessed in the offspring. Overall, a significant sex × treatment interaction effect was observed after prenatal exposure to maternal immune challenge by Poly I:C, indicative of increased global HDAC activity particularly in female offspring from mothers injected with Poly I:C when compared to controls. Results on the levels of specific HDAC isoforms suggested that neither differences in the levels of HDAC1, HDAC2, HDAC3, HDAC4 or HDAC6 could explain the increased global HDAC activity observed in female Poly I:C offspring. In conclusion, we show that Poly I:C administration to pregnant mice alters global brain HDAC, but not DNMT activity in adult offspring, whereas it is still unclear which specific HDAC(s) mediate(s) this effect. These results indicate the necessity for further research on the epigenetic effects of Poly I:C.

## Introduction

Infection during pregnancy and its associated maternal immune response may negatively impact upon offspring brain development [1, 2], thereby increasing the risk to develop psychopathology later in life [3, 4]. The exact mechanisms by which prenatal maternal inflammation impacts upon offspring brain development remain to be elucidated though.

Several studies examined the effects of maternal infection during gestation in animal models using different triggers such as lipopolysaccharide [5, 6], the human influenza virus [3] and polyinosinic:polycytidylic acid (Poly I:C). Poly I:C is a synthetic double stranded RNA frequently used as a viral mimetic that induces the release of pro-inflammatory cytokines [7, 8]. As such, prenatal Poly I:C injection to the dams has been shown to induce various cognitive and behavioural changes in adult offspring [9] including impairments in exploratory behaviour, prepulse inhibition, latent inhibition and spatial working memory [10]. Moreover, moderate to severe cell loss was observed in the adult hippocampal CA1, CA3 and dentate gyrus after prenatal maternal exposure to Poly I:C [11]. As Poly I:C is known to induce a short-lasting immune response, the timing of the maternal inflammatory response can be accurately linked to specific windows during foetal development [11]. Interestingly, when Poly I:C was injected at GD 6 or GD 9, impaired latent inhibition was observed in the offspring, whereas this effect was less pronounced when Poly I:C was injected at GD 13 and was even absent when injection occurred at GD 17 [12]. In the literature, most of the studies use Poly I:C injection to the pregnant dams at GD 9, which roughly corresponds to the end of the first trimester of human pregnancy. Prenatal maternal infection during this period has mainly been associated with the development of schizophrenia [13–15]. However, prenatal maternal immune challenge with Poly I:C at GD 17 has been linked primarily to the development of depressive-like behaviour in the offspring [16, 17].

Despite the fact that this field of research has received increased attention, the exact mechanisms behind the association between maternal infection during pregnancy and altered brain development in the offspring have still not been elucidated. One likely candidate mechanism involved is epigenetic programming, which is known to play a critical role during fetal development. Furthermore, epigenetic processes modulate several functions in the adult nervous system, such as adult neurogenesis, synaptic plasticity, as well as cognition and emotionality [18]. The most well-known epigenetic processes include DNA methylation, histone modifications and noncoding RNAs. Epigenetic changes result in chromatin reorganization and thus regulate gene expression during e.g. cellular differentiation. More specifically, histone deacetylation, which generally leads to the compaction of chromatin and transcriptional repression, has recently been linked to the pathophysiology of various psychiatric disorders. For example, changes in histone acetylation have been reported in animal models of depression and stress exposure [19]. In addition to histone modifications, DNA methylation has also been shown to be involved in the development of depressive-like behaviours [20], whereas both histone deacetylase (HDAC) and DNA methyltransferase (DNMT) inhibitors have been reported to exert antidepressant effects [19, 20]. In view of prenatal infection, hypoacetylation of histones H3 and H4 has been observed in the cortex of juvenile offspring prenatally exposed to Poly I:C [21]. Moreover, HDAC inhibitors are known to impact upon the transcriptional regulation of cytokines, immunologic signaling pathways and the inflammatory response [22]. To date, the epigenetic effects of prenatal maternal Poly I:C exposure at GD 17-which, as stated above, links more closely to the development of a depressive-like phenotype- have not yet been studied.

In the present study, we examined the brain-specific global epigenetic effects of prenatal maternal immune challenge using viral mimetic Poly I:C at GD 17 in C57BL/6JRccHsd mouse offspring. We hypothesize that prenatal maternal Poly I:C exposure would lead to increased global HDAC and DNMT activity, which represents a molecular switch modulating the alterations in offspring brain development in response to prenatal maternal inflammation.

## Materials and methods

### Animals

Experiments were conducted in accordance with permissions from the government and the veterinarian administration of Oberbayern (AZ: 55.2-1-54-2531-61-10). This study used C57BL/6JRccHsd mice obtained from Harlan Laboratories (Eystrup, Germany). Mice were housed 4 per cage in single-sex groups in individually ventilated cages (IVC; cage sizes 480 × 375 × 210 mm [depth × width × high]) under specific pathogen free (SPF) conditions and maintained on a 12/12 h light–dark cycle (light on at 12AM) and temperature-controlled environment (relativity humidity 55 ± 5 %; temperature: 22 ± 2 °C: room air exchange rate: 15). Breeding food (Ssniff, Germany) and water were allowed ad libitum. Cages were changed every week. After transporting the mice to the local animal facilities, the habituation period lasted 3 weeks. During this period, mice were subcutaneously injected with a transponder for identification purposes. For mating, a male was added to a cage with a female for a period of 3 days. After mating, females were housed individually. Three weeks after birth, offspring were weaned and separated by sex (4 mice/cage). Offspring were chipped when they were 6 weeks old to facilitate their identification. A maximum of 2 mice/sex/litter were used to prevent litter effects [23].

### Mating and induction of prenatal infection

Approximately 8 weeks after birth, male and female offspring were put together for 3 days. At GD17, pregnancy was confirmed by visual inspection of the dam in combination with weight gain analysis (increase between day of mating and the day of the injection). Pregnant mice were randomly divided into 2 groups [Poly I:C; n = 5] and phosphate buffered saline [PBS; n = 6]). Dams were injected intravenously with 5 mg/kg Poly I:C [potassium salt, Sigma Aldrich (Germany)] dissolved in sterile water or an equivalent volume of PBS. The Poly I:C dose chosen has been consistently shown to cause behavioural abnormalities and to induce changes in cytokines in rats and mice [10, 24, 25]. Therefore, it is considered a valid animal model of maternal immune activation in relation to psychiatric disorders. Following injection, mice were observed daily until delivery.

### Enzymatic HDAC and DNMT activity

At an age of thirteen weeks, brains from 8 male and 9 female mice prenatally exposed to Poly I:C, and 9 male and 9 female offspring from PBS-treated dams were used for epigenetic analysis. For this purpose, mice were anesthetized with a single intraperitoneal injection of Ketamin (Pfizer, Germany) and Xylacin (Bayer, Germany). 0.7 ml 10 % Ketamin and 0.3 ml 2 % Xylacin were mixed and administered at a dose of 10 ml/kg. Afterwards, brains were collected and stored at −20 °C in RNA-Later (Invitrogen, Germany). Half of the brain was used to extract nuclear proteins using a Nuclear Extract Kit (Active Motif, Belgium) and the activities of various HDAC and DNMT enzymes were analyzed with the HDAC Fluorescent Assay Kit and DNMT Activity/Inhibition Assay (Active Motif, Belgium). The fluorescence/optical density was detected with a PolarStar OPTIMA microplate fluorimeter (BMG Labtech).

### Western blot

In order to elucidate which specific HDAC enzyme(s) would mediate the observed increase in overall HDAC activity (see below), a western blotting experiment was performed. For this purpose, the other half of each brain was crushed while frozen and stored at −80 °C. Tissue was subsequently mechanically homogenized in the Mini-Beadbeater™ (Biospec, The Netherlands) using lysis buffer (PBS, 1 % Igepal CA-630^®^, 0.1 % triton, 1 % glycerol, 1 mM EDTA, 1 mM EGTA) and protease inhibitors (Complete Protease Inhibitor Cocktail, Roche Diagnostics, The Netherlands) in Milli-Q. Concentration of total protein was measured using the Lowry protein assay (Bio-Rad, The Netherlands). Proteins dissolved in sample buffer (1 M Tris HCl pH 6.8, 75 % glycerol, SDS, β-mercaptoehtanol, bromophenol blue in Milli-Q) were denatured by boiling at 100 °C for 7 min. Thirty microgram of protein per lane, in case of HDAC4 and 6, and 100 µg for HDAC1, 2 and 3 was separated by 10 % SDS-polyacrylamide gel electrophoresis (SDS-PAGE) for 75 min at 150 V and transferred to a nitrocellulose membrane during 90 min at 100 V using a Bio-Rad Laboratories Western Blotting system. Next, membranes were rinsed once in PBS and blocked in 1:1 Odyssey Blocking Buffer:PBS (LICOR Biosciences, NE, USA) for 1 h at room temperature. Following the blocking of the membranes, they were incubated overnight at 4 °C with the respective monoclonal mouse anti-mouse HDAC1 (1:350), HDAC2 (1:500), HDAC3 (1:350) and monoclonal anti-rabbit HDAC4 (1:500) contained in the HDAC Antibody Sampler Kit (#9928, Cell Signaling Technology, Inc.) or the monoclonal anti-rabbit HDAC6 (1:500) (#7612, Cell Signaling Technology, Inc.) and 1:1000 diluted monoclonal mouse anti-β-actin (sc-81178, Santa Cruz Biotechnology) diluted in either 1:1 Odyssey Blocking Buffer:PBS (HDAC1, 3, 4) or 1:1 Blocking buffer:PBS + 0, 2 % Tween-20 (HDAC2 and 6). After incubation with the primary antibody, membranes were rinsed once with PBS:Tween and twice with PBS and then incubated with the corresponding secondary antibody, i.e., Alexa Fluor^®^ 680 donkey anti-mouse (1:10000) IgG or Alexa Fluor^®^ 800 goat anti-rabbit IgG (1:5000; LI-COR Biosciences) diluted in 1:1 Blocking buffer:PBS for 1 h at room temperature. Membranes were rinsed again as explained before and bands were visualized by enhanced chemiluminescence detection using an Odyssey Scanner and the Odyssey Infrared Imaging System v2.1 software (LI-COR Biosciences). Bands were quantified with the ImageJ software (NIH, USA) using mean intensity and normalizing for β-actin expression.

### Statistical analysis

The effects of prenatal maternal Poly I:C exposure on enzymatic HDAC and DNMT activity and protein levels of each HDAC isoform were first subjected to a two-way analysis of variance (ANOVA; treatment × sex) with IBM SPSS Statistics version 20 (SPSS Inc., USA). Significant interaction effects were analyzed in more detail using post hoc LSD tests. The level of significance was set at p < 0.05 in all cases. Graphs were designed with GraphPad Prism 6.

## Results

### Global enzymatic activity

**Fig. 1:**
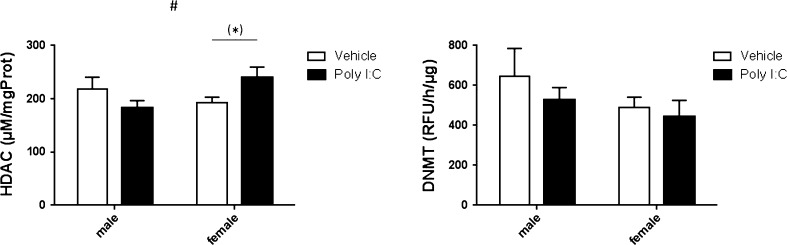
Modulation of HDAC global brain activity in mouse adult offspring by prenatal Poly I:C exposure. Bars represent means + SEM, # p < 0.05 (ANOVA, sex × treatment), (*) p = 0.064 (LSD). Vehicle male n = 9, vehicle female n = 9, Poly I:C male n = 8, Poly I:C female n = 9. For total DNMT activity no effects were detected (F3,31 = 1.676, F3,31 = 0.826, F3,31 = 0.159; all p > 0.05)

Regarding HDAC activity, a significant sex x treatment interaction effect was found (F_3,31_ = 5.477; p < 0.05) (see Fig. 1). Post hoc LSD tests indicated that HDAC activity tended to be increased in female offspring from mothers injected with Poly I:C (p = 0.064) compared to those injected with PBS. No overall effects were found for sex or treatment (F_3,31_ = 0.884 and F_3,31_ = 0.110 respectively; both p > 0.05). For total DNMT activity, no significant effects were detected (F_3,31_ = 1.676, F_3,31_ = 0.826, F_3,31_ = 0.159 for sex, treatment and their interaction, respectively; all p > 0.05) (see Fig. 1).

### HDAC isoforms

**Fig. 2:**
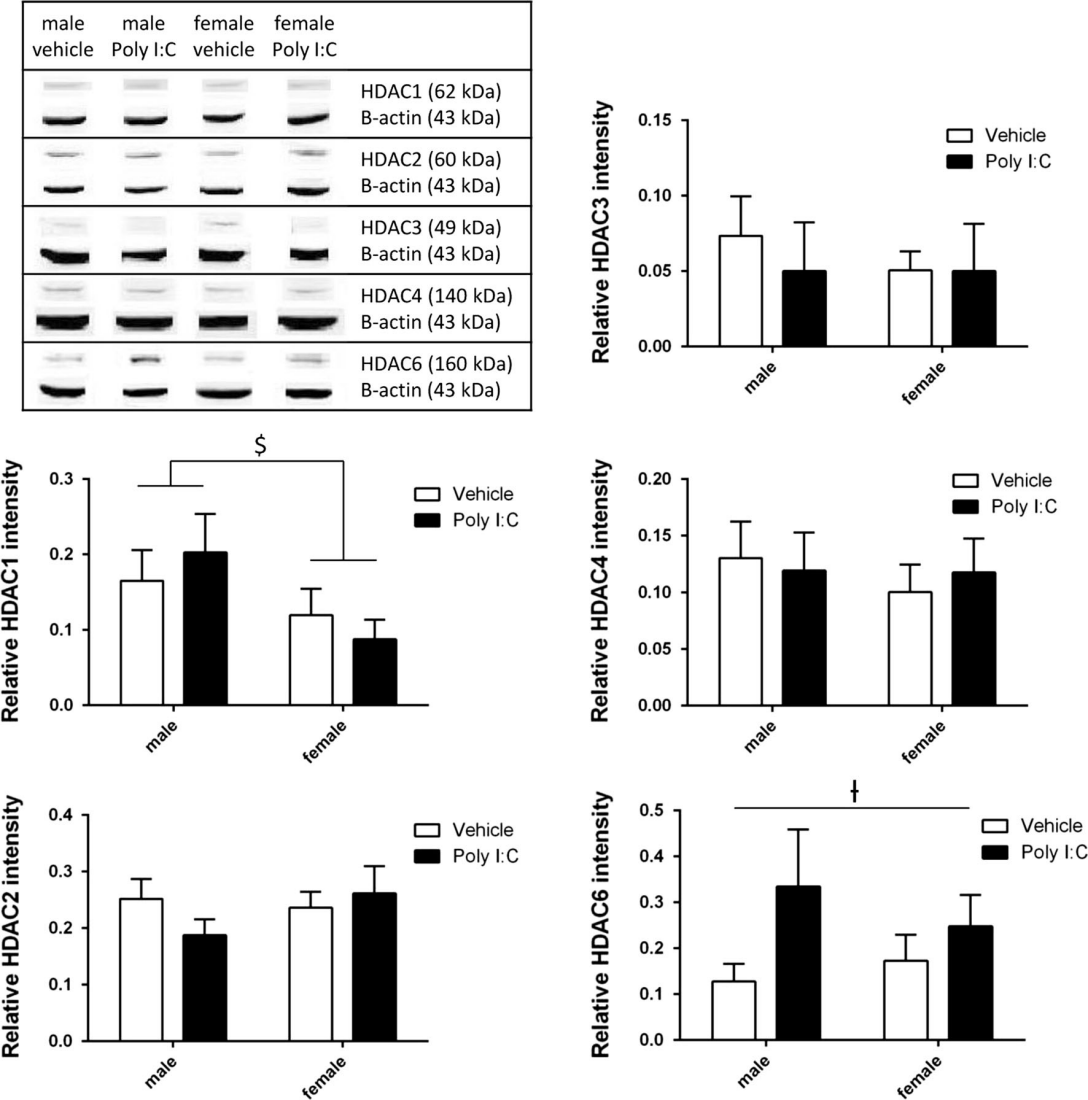
Levels of HDAC1, HDAC2, HDAC3, HDAC4 and HDAC6 proteins in brain homogenates and corresponding quantification. Bars represent means + SEM, ^$^p < 0.05 (ANOVA, sex), ^†^p < 0.1 (ANOVA, treatment). HDAC1-5: Vehicle male n = 9, vehicle female n = 9, Poly I:C male n = 8, Poly I:C female n = 9. HDAC 6: Vehicle male n = 9, vehicle female n = 9, Poly I:C male n = 6, Poly I:C female n = 9

Overall, a sex effect was observed with reduced HDAC1 levels in female versus male mice (F_3,31_ = 4.389; p < 0.05). Furthermore, a tendency for an overall treatment effect was shown for HDAC6 with increased protein levels in Poly I:C offspring when compared to control offspring (F_3,29_ = 4.061; p = 0.053). No effect was found for HDAC2, HDAC3, and HDAC4 (all F_3,31_ < 3; p > 0.05) (see Table 1 and Fig. 2).

**Table 1:** Mean and SEM for each condition for the correspondent HDAC

	Male vehicle		Male Poly I:C		Female vehicle		Female Poly I:C		Effects
	M	SEM	M	SEM	M	SEM	M	SEM	
HDAC1	0.1655	0.0402	0.2027	0.0510	0.1195	0.0350	0.0875	0.0264	Sex effect p < 0.05
HDAC2	0.2518	0.0353	0.1874	0.0285	0.2362	0.0278	0.2620	0.0475	
HDAC3	0.0735	0.0262	0.0502	0.0323	0.0506	0.0125	0.0501	0.0313	
HDAC4	0.1303	0.0322	0.1194	0.0335	0.1005	0.0241	0.1178	0.0296	
HDAC6	0.1277	0.0386	0.3348	0.1242	0.1731	0.0563	0.2479	0.0684	Treatment effect p < 0.1

## Discussion

In the present paper, we report that prenatal maternal Poly I:C exposure in the mouse modulates global brain HDAC activity, but not DNMT activity in adult offspring. HDAC activity was particularly increased in female offspring exposed to prenatal maternal Poly I:C when compared to controls, suggesting that epigenetic programming may, at least in part, mediate the long-term effects of prenatal maternal infection.

Exposure to pathogens triggers an inflammatory response with concomitant up-regulation of pro-inflammatory cytokines. Interestingly, next to the fact that HDACs were shown to regulate gene transcription of cytokines [26, 27], cytokines have recently also been shown to regulate the activity of HDACs. For example, activation of pro-inflammatory cytokines, such as tumour necrosis factor-α (TNF-α) and interferon-γ (IFN-γ), produces HDAC1 ubiquitinization and proteosomal degradation [27]. Apart from the modulation of innate immune system function, HDACs also regulate adaptive immunity [28]. Depletion of both HDAC1 and HDAC2, specifically in B cells leads to blocking of early B cell development [29]. Over the last years, increased evidence linked histone acetylation to the neurobiology and pharmacology of mood disorders [30]. For instance, chronic defeat stress resulted in an impaired behavioural response and in the downregulation of brain-derived neurotrophic factor transcripts III and IV, which was reversed by imipramine. Interestingly, this antidepressant also increased histone acetylation at these promoters and was associated with a downregulation of HDAC5 [31]. Vice versa, HDAC inhibitors have also been shown to possess antidepressant properties [32, 33]. For example, administration of the HDAC inhibitor sodium butyrate alone or in combination with fluoxetine decreased immobility scores in the tail suspension test, a measure for behavioural despair, in mice [34]. However, these studies used naïve mice that were not exposed to a prenatal immune challenge. As mentioned above, the effects produced by prenatal maternal infection are depending on the timing of exposure during gestation and their behavioural expression may also depend on the age of the offspring. For instance, after Poly I:C injection at GD 9, global histone H3 and H4 hypoacetylation was shown in in the cortex of juvenile but not adult offspring [21]. Our results are partially consistent with this study, e.g. we found that HDAC activity was increased in offspring that were exposed to prenatal maternal Poly I:C (at GD 17), an effect that seemed to be particularly pronounced in females. However, our findings were observed in adult offspring, whereas Tang and colleagues detected hypoacetylation only in juvenile offspring, a difference that may be explained by the different timing of prenatal maternal Poly I:C exposure.

We hypothesized that the observed increase in global HDAC activity in females, which were prenatally exposed to maternal Poly I:C, would be reflected in enhanced expression of one of the HDAC proteins assessed. However, the western blot showed that none of the HDAC enzymes examined was present in significantly higher levels in Poly I:C versus control female offspring. Although we observed a treatment effect with increased HDAC6 in offspring of mice treated with Poly I:C compared to controls, this effect seemed to be more pronounced in male—and not female—offspring, which does not match the global HDAC activity data.

In the brain, HDAC6 is involved in protein aggregate elimination, in neuronal oxidative stress and in the mitochondrial transport [35]. Interestingly, HDAC6 deficiency in mice resulted in hyperactivity and reduced anxiety, and depression-like behavior. Similarly, administration of an HDAC6 inhibitor also had an antidepressant-like effect, suggesting a role for HDAC6 in the expression of emotional behaviours [36]. As commented above, prenatal maternal Poly I:C exposure has been associated with the development of depressive-like behaviour in the offspring. Interestingly, we observed that HDAC6 protein levels tended to be increased in Poly I:C offspring when compared to control offspring. Altogether, though purely speculative, these data suggest a role for HDAC6 in mediating the association between prenatal maternal infection and the development of depression in the offspring. Thus, further research investigating this possible role of HDAC6, would be of great value.

In addition, we observed a sex effect with reduced HDAC1 levels in female mice compared to males. Regarding brain functions, HDAC1 is reported as the molecular switch between neuronal survival and death [37] and being considered as a negative regulator of fear extinction in mice [38] and as a negative regulator of mood [39]. No studies have examined sex differences in HDAC1 function in this respect. Thus, future research should address possible variations in HDAC1 levels between sexes.

A feasible explanation for the discrepancy observed between the global HDAC activity and the protein levels of several HDAC isoforms in the present study is that one or more other HDAC isoform(s) than those assessed using western blotting is/are involved in mediating the observed increased in global HDAC activity. HDACs are part of a large family of proteins, with 11 members identified to date. We examined HDAC1, HDAC2, HDAC3, HDAC4 and HDAC6, because of the evidence showing their association with mental (dys)function [36, 38, 40–42]. A viable candidate that may contribute to the effect found in HDAC global activity is HDAC5, which has been shown to be involved in inflammation, specifically in activated monocytes and macrophages [43]. Moreover, decreased HDAC5 levels in the hippocampus has been associated to the antidepressant effects of imipramine in mice models, as explained above [31]. We initially included HDAC5 in the present study, but after trying numerous different antibodies in several organ tissues, we were not able to detect a specific band indicating HDAC5. Another, completely different possible explanation for the observed discrepancy may lie in the fact that an increased enzymatic activity does not necessarily imply an increase in the level of the associated protein(s). Clearly, this issue awaits further investigation.

## Conclusions

Altogether, this study indicates that prenatal maternal Poly I:C exposure seems to increase global HDAC, but not DNMT, activity particularly in female offspring. Evidently, it would be of great value to further explore the exact role of epigenetic programming in response to prenatal exposure to maternal immune challenge using Poly I:C in order to understand the mechanism underlying the association between prenatal infection/inflammation and adult psychopathology.

### Limitations

It should be noted that mice were anesthetized in this study in order to comply with the approval of the ethics committee. Although the time window between exposure and actual euthanasia was extremely short in our case, we cannot fully exclude the possibility that the use of anesthesia may have influenced epigenetic processes [44]. In addition, it should be noted that for assessing both HDAC levels and activity, whole brain homogenates were used. It would evidently be of additional interest to evaluate specific brain areas and cell-type-specific epigenetic changes. Last, the dose of 5 mg/kg Poly I:C used in the experiment has been consistently shown to be effective in inducing changes in cytokines and alterations in the behaviour of mice and rates. It should be noted however that evidence is lacking on how such a Poly I:C dose captures the principle of human prenatal maternal infection.
